# Beyond the Intestinal Celiac Mucosa: Diagnostic Role of Anti-TG2 Deposits, a Systematic Review

**DOI:** 10.3389/fmed.2014.00009

**Published:** 2014-05-02

**Authors:** Simona Gatti, Matilde Rossi, Simona Alfonsi, Alessandra Mandolesi, Giovanni Cobellis, Carlo Catassi

**Affiliations:** ^1^Department of Pediatrics, Università Politecnica delle Marche, Ancona, Italy; ^2^Department of Surgical Pathology, Università Politecnica delle Marche, Ancona, Italy; ^3^Department of Pediatric Surgery, Università Politecnica delle Marche, Ancona, Italy

**Keywords:** intestinal deposits, anti-tissue transglutaminase 2, celiac disease, potential celiac disease, dermatitis herpetiformis, immunofluorescence

## Abstract

**Aim:** To review the existing literature on the role and significance of intestinal transglutaminase 2 immunoglobulin A deposits (TG2 deposits) in patients with overt celiac disease (CD), potential celiac disease (PCD), and other autoimmune or gluten-related conditions.

**Methods:** We conducted a systematic review of studies published in English, evaluating presence and characteristics of TG2 deposits in subjects with overt CD, PCD, gluten-related diseases [dermatitis herpetiformis (DH), gluten-ataxia (GA)], autoimmune disorders (type-1 diabetes), and other conditions. Studies were identified through a MEDLINE search (1950–2013).

**Results:** Twenty-three studies were included in the review. Eleven studies were performed in children. Overall TG2 deposits were present in 100% of adults with overt CD, while in children prevalence ranged from 73.2 to 100%. Six studies with an established definition of PCD were considered, prevalence of deposits ranging from 64.7 to 100%. A single study followed-up PCD patients with repeated biopsies and identified presence of intestinal deposits as the best marker to reveal progression toward villous atrophy. Two studies investigated presence of deposits in DH, reporting prevalence between 63 and 79%. A single study documented TG2 deposits in 100% of patients with GA. In children with type-1 diabetes (T1D), positivity of intestinal TG2 deposits ranged from 25 to 78%.

**Conclusion:** Transglutaminase 2 IgA deposits seem to be a constant feature in overt CD patients and are frequently detectable in other gluten-related conditions (DH and GA). The vast majority of PCD patients express TG2 deposits at the intestinal level, but no sufficient data are available to exactly define their prognostic role as a marker of evolution toward overt CD. The frequent finding of TG2 deposits in the intestinal mucosa of patients with T1D is an interesting observation deserving further evaluation.

## Introduction

Celiac disease (CD) is a chronic, immune-mediated enteropathy triggered by the ingestion of gluten containing grains in genetically susceptible individuals, expressing the HLA-class 2 molecules DQ2 or DQ8 (1). Clinical features vary greatly from asymptomatic to classical or atypical presentation. Gluten-induced small-bowel (SB) mucosal histological damage develops gradually, from lymphocytic infiltration of the epithelium to crypt hyperplasia and further to villous atrophy (2). Histological confirmation of CD can be challenging, as related to the quality of the biopsy specimens (3) as well as the patchiness of the intestinal lesions (4). Furthermore, similar SB mucosal abnormalities can be found in other conditions such as giardiasis, viral infections, food allergy, and autoimmune enteropathy.

Potential celiac disease (PCD) is a well-known condition, characterized by a positive serology for CD with an architecturally normal intestinal mucosa. Timing of progression toward classical CD has not clearly been established in PCD, although a recent study indicates that 30% of children with PCD left on a normal diet develop villous atrophy within 4 years (5). In another study performed in an at-risk population (first-degree relatives), 5% of children with PCD developed overt CD within 2 years (6). In this context, the search for a specific marker with a high prognostic value is extremely appealing.

**Figure 1:**
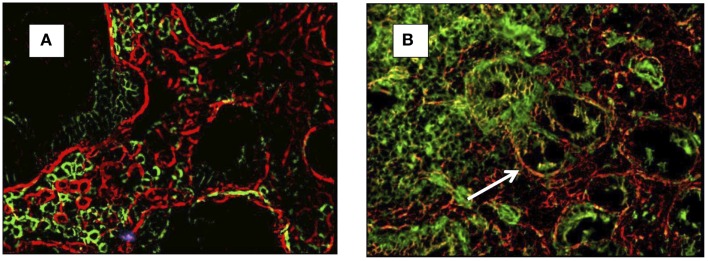
**Small-bowel mucosal immunofluorescence staining of IgA (green) and transglutaminase-2 (red) and co-localization of IgA and TG2 in yellow (arrow)**. In **(A)** a healthy control (no co-localization), in **(B)** a celiac patient with evidence of co-localization (arrow).

It was initially observed that the SB epithelial basement membrane of CD patients contains specific deposits of immunoglobulin A (IgA) (7), which were subsequently shown to be targeted against extracellular transglutaminase 2 (TG2) (8). The original technique to detect the presence of anti-TG2 IgA extracellular deposits was introduced by Karponay-Szabò et al. (8). In the original paper, authors accurately described a method of detecting the co-localization of IgA deposits to TG2 by immunofluorescence (Figures 1A,B). Based on this technique, they demonstrated presence of TG2 deposits in the intestinal mucosa of CD patients, PCD patients, and subjects with dermatitis herpetiformis (DH).

So far several studies investigated the presence and the diagnostic role of TG2 IgA deposits in adults and children with either overt CD or PCD. Intestinal deposits have been found to be a constant feature in overt CD at diagnosis, while their intensity seems to decrease on a gluten-free diet (GFD). Their role in PCD is less clear, with some studies reporting a positive value in predicting an intestinal damage. Furthermore intestinal deposits have been described in other gluten-related disorders (DH and GA) and in other autoimmune conditions, such as type-1 diabetes (T1D).

The determination of intestinal IgA deposits requires a fresh biopsy sample to be stored in liquid nitrogen. It is a time consuming and expensive analysis, requiring an expert pathologist in a specialized center. In consideration of such limitation and the growing amount of data now available, the exact diagnostic role of TG2 IgA deposits needs to be clarified. In this article, we provide a systematic review of the existing literature on the diagnostic value of intestinal deposits in CD and other gluten-related disorders, aiming to answer the question whether this test can be used as an early marker of disease progression in PCD.

## Materials and Methods

Before starting the search and review process, we defined the review protocol, specifying the research question, inclusion and exclusion criteria, search strategies, quality criteria, methods for extracting related data, and statistical methods.

All studies (including cross-sectional, cohort, case–control, and case-series) were considered eligible. Case reports, abstracts presented at meetings, and articles not published in English language were excluded. Our search was focused on studies recruiting patients of all ages with overt CD (associated with some degree of villous atrophy at the SB biopsy), PCD, other gluten-related diseases (gluten-sensitivity, GA, and DH), or other autoimmune conditions [T1D, inflammatory bowel disease (IBD), autoimmune thyroiditis], where the prevalence of intestinal anti-TG2 deposits has been investigated, as a primary or secondary outcome. Overt CD was defined according to the European Society of Pediatric Gastroenterology, Hepatology and Nutrition (ESPGHAN) criteria (9). PCD was defined as the positivity of serological markers [anti-transglutaminase (TTG) and/or anti-endomysium (EMA) antibodies] in presence of normal SB histology or minimal histological changes (Marsh 0–1). Studies giving ambiguous definitions of PCD or considering PCD patients together with gluten-sensitive or latent CD patients were not considered. No publication date or publication status was imposed.

Relevant studies were identified through electronic databases and scanning reference lists of eligible studies. Our search was applied to the Medline database using PubMed by combining key words for gluten-related disorders (CD, PCD, gluten intolerance, gluten-sensitivity, GA, DH, autism, type-1 diabetes) and search terms for intestinal deposits (intestinal deposits, IgA intestinal deposits, anti-TG2 intestinal deposits, intestinal anti-tissue transglutaminase, immunofluorescence). All studies were published between 1950 (start of Medline) and December 2013. To ascertain the validity of the eligible studies, the study design, the size, and representativeness of the study population (i.e., the presence of selection bias), the validity of outcomes (risk of confounding or bias), and the quality of the statistical analysis were taken into account.

The following informations were extracted from each study: (1) characteristics of participants (number, age, diagnosis and method of diagnosis, diet, length of diet); (2) serological markers [TTG, EMA, anti-gliadin antibodies (AGA) IgA and IgG]; (3) prevalence, type, and features of intestinal deposits (anti-TG2, anti-endomysium and AGA, intensity, submucosal or perivascular, homogeneous or heterogeneous pattern).

## Results

### Study characteristics

Overall we identified 25 eligible studies. Two of them were subsequently excluded because prevalence of TG2 deposits was not reported in the results in one and intestinal IgA deposits instead of TG2 deposits were considered in the other (10, 11). We found 18 studies performed in celiac (overt and potential) patients, 9 of them in pediatric groups. The remaining five studies were focused on children with T1D and cerebral palsy and adult with GA, DH, and gluten-sensitivity. Table 1 summarizes the features of the 23 selected studies.

**Table 1:** **Summary of included studies evaluating presence and significance of intestinal deposits in celiac disease and other groups of patients**.

No.	Source (first author)	Study design	Number of participants	Group 1 (*n*)	Inclusion criteria	Group 2 (*n*)	Inclusion criteria	Controls (*n*)	Age	Outcomes
1	Tosco (5)	O, Cohort, P	106	106	PCD	–	–	–	6 years, 8 months	Histological and serological follow-up study in children with PCD
2	Korponay-Szabo (8)	O, Cohort	33	10	Developing CD	11	CD	12	8 years, 1 month	Prevalence of intestinal and extra intestinal TG2 IgA deposits in early stage CD and CD
3	Salmi (12)	O, C.S.	427	223	Untreated CD	66	PCD	138	45 years	Comparison between conventional and new histological parameters (including TG2 deposits) in diagnosis of CD and PCD
4	Salmi (13)	O, Cohort, P	75	25	EMA− Marsh 1	25	PCD	25	39 years	Role of TG2 IgA deposits in detection of early developing CD
5	Koskinen (14)	O, C.S.	254	71	CD on short term GFD	105	CD on long term GFD	78	47 years	Prevalence of TG2 IgA deposits in CD at diagnosis and after GFD
6	Koskinen (15)	O, C.S.	103	48	LCD, PCD	13	CD	42	37 years	Diagnostic value and gluten-dependency of TG2 IgA deposits in overt and mild enteropathy CD
7	Kaukinen (16)	C, E	93	41	Suspected CD (normal villi, ↑γδ+ IELs)	18	CD	34	46 years, 9 months	Prevalence of TG2 IgA deposits in early stage CD before and after dietary intervention
8	Maglio (17)	O, C.S., R	344	144	Untreated CD	109	PCD	91	6 years, 3 months	Prevalence of TG2 IgA deposits in CD and PCD
9	Maglio (18)	O, C.S.	203	104	CD <2 years	179	CD > 2 years	18	15 months	Prevalence of TG2 IgA deposits in CD children <2 years, negative for serum anti-TG2 Ab
10	Tosco (19)	O, C.S.	40	12	CD	28	PCD	39	7 years	Comparison of Ab secretion to culture supernatants and TG2 deposits in CD and PCD
11	Kurppa (20)	O, Cohort, P	76	42	Untreated CD	17	PCD	17	11 years	Evaluation of deposits at diagnosis in CD, PCD, and controls
12	Salmi (21)	O, C.S., P	193	22	CD, EMA+	151	CD, EMA−	20	47 years	Comparison of clinical and histological features (including TG2 deposits) in EMA negative and positive CD
13	Koskinen (22)	O, C.S.	65	28	Responsive CD	27	Non-responsive CD	10	48 years	Role of TG2 deposits in distinguishing non-responsive CD from other enteropathies
14	Koskinen (23)	C, E.	23	13	CD on GFD + oats challenge	10	CD on GFD + gluten-challenge	–	13 years	Response of TG2 deposits to oats challenge
15	Salmi (24)	O, C.S.	74	47	Untreated DH	27	DH on GFD	–	47 years	Prevalence of TG2 IgA deposits in DH and relation to GFD
16	Hadjivassiliou (25)	O, C.S.	36	97	GA, other ataxia	10	CD	10	59 years, 6 months	Deposition of brain and intestinal TG2 IgA deposits in patients with GA
17	Stenberg (26)	O, C.S.	16	16	CP with CD	–	–	–	11 years	Detection of early signs of CD in CP
18	Maglio (27)	O, C.S.	100	33	T1D on GCD	12	Untreated CD	55	11 years	Prevalence of TG2 IgA deposits in children with T1D
19	Borrelli (28)	O, C.S.	25	16	IgA–D, Marsh 0–1; nine non-CD; seven PCD (IgG pos)	9	IgA–D; CD (Marsh 2–3)	16	7 years, 1 month	Detection of TCR γδ IELs and TG2 IgM deposits in IgA–D subjects
20	Ruuskanen (29)	O, Cohort, P	2815	49	AGA+, tTG−, HLA+, no CD	–	–	52	69 years	Detection of minor inflammatory changes in AGA persistently positive non-CD patients
21	Tosco (30)	O, C.S.	91	39	PCD	18	Serum Ab negative, IEL+	34	7 years, 1 month	Prevalence of TG2 IgA deposits in PCD
22	Stenman (31)	O, C.S.	31	5	Untreated CD	20	CD on GFD	6	54 years	Correlation between Ab secretion to culture supernatants and TG2 deposits
23	Tack (32)	C, E	16	7	CD gluten-challenge + placebo	7	CD gluten-challenge + EN PEP	–	55 years	Evaluation of safety and immunogenic effects of AN-PEP in CD

*C, comparative; O, observational; E, experimental; P, prospective; R, retrospective; CD, celiac diseases; PCD, potential celiac disease; LCD, latent celiac disease; GFD, gluten-free diet; T1D, type-1 diabetes; CP, cerebral palsy; GA, gluten-ataxia; AN-PEP, *Aspergillus niger* prolylendoprotease; IgA D, IgA deficiency; DH, dermatitis herpetiformis; EMA, anti-endomysium antibodies; tTG, anti-transglutaminase antibodies*.

### Intestinal deposits in CD at diagnosis

Ten studies investigating the presence of intestinal TG2 deposits in patients with overt CD at diagnosis were identified and five were performed in children (median age 6.5 years). The included studies involved a total of 863 CD patients, presence of deposits was investigated only in 81.7% of cases. Results of the included studies are summarized in Table 2.

**Table 2:** **Summary of the included studies evaluating the presence of intestinal TG2 deposits in overt celiac disease (CD)**.

Reference (first author)	Age (years), median (range)	Overt CD (*n*)	TG2 IgA deposits: prevalence	Diagnostic value (%)
Korponay- Szabo (8)	78 (4.4–32)	10	10/10	SE: 100
				SP: 100
Salmi (12)	42 (16–81)	223	35/35	SE: 100
				SP: 100
Koskinen (14)	47 (4–79)	261	261/261	SE: 100
				SP: 82
Koskinen (15)	47 (28–68)	13	13/13	SE: 100
				SP: 95.9
Kaukinen (16)	47 (22–68)	18	6/6	SE: 100
				SP: 100
Maglio (17)	63 (6 months–16 years)	144	138/144	SE: 95.8
				SP: 87.9
Tosco (19)	7	12	12/12	SE: 100
				SP: 80
Maglio (18)	<2, 15 months	<2 years: 56	<2 years: 41/56	SE: 73.2
	>2, 7 years	>2 years: 40	>2 years: 40/40	SE: 100
				SP: ne
Kurppa (20)	10 (1–15)	42	42/42	SE: 100
				SP: ne
Salmi (21)	55 (20–79)	22 EMA−	18/18	SE: 100
	40 (16–81)	22 EMA+	17/17	SP: 100

*SE, sensitivity; SP, specificity; ne, not evaluable (no control group)*.

Adults with untreated CD had TG2-targeted autoantibodies deposition in the SB mucosa in 100% of cases (12–16), while there was more variability in the pediatric population. By studying 144 untreated CD children, Maglio and co-workers found that TG2 IgA deposits had a sensitivity of 95.8% in diagnosis of CD (17). The same authors reported a different prevalence of TG2 IgA deposits in younger children (<2 years of age) compared to older groups. They found mucosal deposits in 73.2% of infants, while the detection rate of serum EMA and/or anti-TG2 IgA was 82% in the same population. Conversely all children older than 2 years of age were positive for anti-TG2 IgA intestinal deposits and had serum EMA and circulating TTG antibodies (18). Three pediatric studies showed that deposits had a sensitivity of 100% in untreated CD, confirming adult results (8, 19, 20). Furthermore intestinal TG2-targeted autoantibody deposits had the best sensitivity values in detecting untreated CD with villous atrophy comparing with other CD markers [increased density of villous tip intraepithelial lymphocytes (IELs), increased density of γδ+ IELs, increased density of CD3+ IELs, serum IGA-class autoantibodies] in adult setting (12).

Overall, the mean value of specificity in all selected studies (either adult patients or children) was 94.15% (range 82–100%). Two studies found the intensity of these deposits significantly higher in the celiac patients than in patients with PCD (17, 20). Interestingly Salmi et al. found that in CD patients without serum EMA, TG2 IgA were still deposited and detectable in the SB mucosa in all cases (21). However, the same authors found that the intensity of deposits did not correlate with the severity of the mucosal lesion (21).

### Intestinal deposits in PCD

Several studies investigated the presence and features of intestinal deposits in subjects with PCD, both in children and adults. Six studies included a well-defined group of PCD patients, their main characteristics, and results are summarized in Table 3.

**Table 3:** **Summary of the included studies evaluating TG2 deposits in potential celiac disease (PCD)**.

Reference (first author)	Age, median (range)	PCD (*n*)	PCD: definition	TG2 IgA deposits: prevalence	Diagnostic value (%)	Intensity	Results of follow-up studies and comparison with other markers
Tosco (5)	6 years, 8 months (18 months–16 years)	106	TTG or EMA+, Marsh 0–1	66/102	SE: 64.7 SP: ne	–	At 4 years follow-up, 30.8% develop overt CD. TG2 deposits in the first biopsy was the only marker to predict evolution toward villous atrophy
Salmi (13)	40 years (16–81)	25	EMA+, Marsh 0–1	12/12	SE: 93	–	TG2 had the best SE and SP for detecting early developing CD
					SP: 93	
Maglio (17)	6 years, 4 months (6 months–16 years)	109	TTG+, Marsh 0–1	74/109	SE: 67.9 SP: 87.9	Intensity significantly weaker in PCD compared to CD	–
Tosco (19)	7 years (2–17)	28	TTG+, Marsh 0–1	19/28	SE: 68	–	–
					SP: 80	
Kurppa (20)	11 years (4–17)	17	EMA+, Marsh 0–2	17/17	SE: 100	Less intense in PCD than CD	Seven of eight on a GCD develop overt CD within 2 years
					SP: 100	
Tosco (30)	7 years, 1 month (9 months–17 years, 11 months)	39	EMA or TTG+, normal histology	33/39	SE: 85 SP: 91	–	–

*SE, sensitivity; SP, specificity; PPV, positive predictive value; NPV, negative predictive value; EMA, anti-endomysium antibodies; TTG, anti-transglutaminase antibodies; PCD, potential CD; ne, not evaluable (no control group)*.

In a total of 301 patients with PCD (289 children and 12 adults), the prevalence of intestinal deposits ranged from 68 to 100%. The intensity of deposits was described in four studies, all of them showing that TG2 deposits were significantly less intense if compared to subjects with overt CD (17, 20, 22, 23). Furthermore, four studies detected a patchy distribution of intestinal deposits, characterized by areas with strong staining, with weaker staining areas and areas completely negative (5, 17, 19, 22) Only one prospective study, aiming to investigate the natural history of PCD, followed-up children with PCD with repeated biopsy (5). Evaluation of intestinal deposits was not the primary outcome of the study and power calculation to address the research question was not mentioned in the article. Comparing data from 12 patients that developed villous atrophy and 27 who did not, authors found that only the presence of intestinal deposits in the first biopsy was different in the two groups, speculating that the presence of TG2 deposits could predict the evolution toward villous atrophy.

### Intestinal deposits in CD during follow-up: Prevalence, correlation with other CD markers, and response to gluten-challenge

Five studies specifically addressed the question whether and when TG2 deposits disappear after establishing a GFD. Koskinen et al. compared TG2 IgA deposits after short term (1 year) and long term (2–41 years) GFD in a large cohort of pediatric and adult CD patients. They found that deposits were still positive in 82 and 56%, respectively (14). These results were confirmed in another study in adults, where 28 patients still showed presence of deposits in 75% of cases after 1 year of GFD (24). Interestingly, CD markers were found to disappear in a sequential order during a GFD. TG2 deposits disappeared after normalization of serum autoantibodies, SB villous atrophy, and the densities of CD3+ IELs. The increased density of γδ+ IELs was the only marker that disappeared later than the deposits (14). The same authors analyzed 27 adults with non-responsive CD and found that 6 patients with poor adherence to the GFD had CD autoantibodies in both serum and SB mucosa. In the remaining 21 non-responsive CD patients with good adherence to the GFD, only 4 had serum autoantibodies while 20 (95%) had deposits in the SB mucosa (24). In addition, the intensity of deposits has been shown to gradually decrease during the GFD (14, 15).

Intestinal deposits were also investigated as an early marker of intestinal damage after gluten-exposure. In an interventional study, Koskinen et al. analyzed 23 CD children in clinical, serological, and histological remission, 13 were randomized to undergo an open oats challenge and 10 a gluten-challenge (GC) for 2 years. At baseline, weak-to-moderate TG2 IgA deposits were present in 4 of 13 in the oat challenge group and in 3 of 10 in the GC group. In the oats group, there was no significant change in the intensity of the deposits within 2 years, while in the gluten group the intensity of the deposits clearly increased and decreased again on a GFD. The intensity of deposits correlated well with serum TG2-antibody levels and the severity of SB mucosal villous damage (23).

In another study by Kaukinen et al., 41 adults with suspected CD (increased density of mucosal γδ+ IELs but normal villous morphology) were randomized to GFD or GC for a 6 months period. Using a clinical score, 11 out of 41 patients were defined as having a gluten-related disorder (5 in the GC and 6 in GFD group). In this subgroup, TG2 deposits were positive in 10 out of 11 and intensity increased upon GC and again decreased on GFD. Conversely IgA deposits were present in only 3 out of 30 patients with suspected CD with no clinical signs of gluten-sensitivity (16).

### Intestinal deposits in other gluten-related disorders, autoimmune conditions, and IgA deficiency

A few studies investigated the prevalence and significance of intestinal deposits in other gluten-related disorders. In subjects with DH, the presence of cutaneous IgA deposits directed against TG3, as a marker of the disease, is well-established. The first study by Karponay-Szabò et al. investigating the presence of intestinal deposits in CD also enrolled 11 patients with DH (8). TG2 deposits were present in seven of them (63%) who had a normal villous architecture and two of them were negative for circulating EMA. One single study specifically explored the intestinal TG2 deposits in DH detecting them in 79% of untreated DH patients (with moderate or severe intensity) and in 41% of GFD treated DH patients (all with weak intensity). The presence and the intensity of the deposits were strongly correlated with the degree of the intestinal damage (25).

A small study (26) reported the presence of anti-TG2 intestinal deposits in 100% of nine patients with GA, and the lack of TG2 deposits in a group of patients with other causes of ataxia and positivity for serum AGA. Stenberg et al. failed to demonstrate an increased prevalence of intestinal TG2 deposits in children with cerebral palsy and elevated levels of CD-related sero-markers (AGA or TTG). In this group of 16 patients, only 1 showed IgA co-localizing with TG2 in the SB mucosa, suggesting CD at an early stage (27).

Interestingly, a high percentage of children with T1D on a normal diet presented with mucosal deposits, irrespective of the presence of circulating TTG antibodies. TG2 intestinal deposits were found in 78 and 58% of T1D children with circulating anti-TTG or not, respectively. Authors further examined molecular features of TG2 deposits, showing that only in the T1D group with positivity of serum TTG, the TG2 deposits had the same molecular characteristics found in CD-TG2 deposits. Particularly the serum-positive T1D patients showed a preferential involvement of the heavy-chain variable region-5 (VH5) antibody gene family, as well as in CD patients (28). In another study by Tosco et al., 20 T1D patients were enrolled as a control group and TG2 deposits were detected in 5 of them (25%) (19).

The presence of intestinal deposits was also investigated in CD children with IgA deficiency. For this purpose, immunofluorescence analysis of IgM anti-TG2 deposits in the SB biopsy of 25 CD children with IgA deficiency, 12 untreated CD with normal IgA levels, 9 PCD with IgA deficiency, and 16 healthy controls was described by Borrelli et al. Intestinal IgM deposits were more prevalent in subjects with CD compared to PCD, but did not discriminate accurately CD from PCD in this group with IgA deficiency (29).

### Intestinal deposits in control groups

Overall, 17 studies included a control group, all of them included patients with negative CD sero-markers, that underwent SB biopsy for GI disorders or other symptoms. The final diagnosis of these patients were functional dyspepsia, irritable bowel syndrome, gastro-esophageal reflux disease, multiple food intolerance, cow’s milk allergy, IBD, iron deficiency anemia, failure to thrive, recurrent abdominal pain, dyspepsia, and T1D. Ten studies did not find intestinal TG2 IgA deposits in the control group (8, 12, 16, 18, 20, 21, 24, 26, 29, 30). Conversely, six studies detected TG2 IgA deposits in a minority of control subjects with a prevalence ranging from 5 to 20%. Tosco et al. found that 3/34 (8.8%) children with normal villous morphology presented TG2 IgA deposits (22). Maglio et al. identified TG2 IgA deposits with a patchy distribution and weak intensity in 11/91 (12%) children, among these 77% had normal mucosa (Marsh 0, 1) whilst 23% had SB mucosa classified as Marsh 3a, but no serological evidence of CD (17). Tosco et al. enrolled 39 control children, of whom 20 had T1D. Deposits were positive in 6/30 (20%) with a patchy distribution and among these 5/6 had T1D (19). Maglio et al. reported on 28 control patients without CD. Among these, 4/28 (14.2%) had TG2 IgA deposits, of whom 3 were affected with IBD (28). Koskinen et al. described the presence of deposits in adult controls in 14/78 (18%) with a weak, often patchy distribution (14). In another study, the same authors enrolled 42 control patients with suspicion of CD but no evidence of villous atrophy in two consecutive biopsy samples. HLA DQ2 or DQ8 were present in 19/42 and 4 patients presented CD serum antibodies at the onset, therefore they have to be considered potential. TG2 IgA deposits were present initially in 1/22 (5%) and in 3/39 (8%) patients on a gluten containing diet in the follow-up biopsy (15). In an *in vitro* study by Stenman et al. (31), authors did not find EMA antibodies in organ culture supernatants derived from non-CD biopsies, but they did not specifically search intestinal deposits in the control group.

### Comparison between intestinal deposits and antibodies secreted into the culture supernatant

Two studies compared the secretion and the deposition of TG2 antibodies at the intestinal level (19, 31). Tosco et al. compared the detection of mucosal deposits to the measurement of antibodies secreted into culture supernatant. In overt CD patients, either TG2 deposits or antibodies secretion in the supernatant (higher than the cut-off value) were detectable in 100% of patients, with no differences when samples were cultured with medium alone or after 24 h of P31–43 or peptic tryptic gliadin digest (PTG). In PCD, the presence of deposits was 67% at baseline and 60 and 90% after 24 h incubation with medium alone and P31–43 or PTG, respectively. Conversely, 96.4% of PCD had IgA antibodies in the supernatant higher than the cut-off (with medium alone) and 92% after 24 h P31–43 or PTG *in vitro* stimulation. In controls, the baseline prevalence of deposits (20%) decrease after culture with medium (7%) and increase after *in vitro* gluten stimulation (36%). In the same group of controls, production of antibodies in the supernatant was 7 and 5% after culture with medium and 24 h P31–43 or PTG incubation. Therefore, authors found the measurement of anti-TG2 in culture supernatants to be more sensitive and specific than the detection of mucosal deposits to reveal mucosal production of anti-TG2 antibodies in CD, showing a sensitivity of 97.5 versus 77.5% and a specificity of 92.3 versus 80% of anti-TG2 in supernatant and mucosal deposits detection, respectively (19).

Stenman et al. demonstrated that only biopsies derived from patients on a short-term GFD and still having positive intestinal TG2 IgA deposits were able to secrete EMA into the culture supernatant, and speculated that autoantibody secretion in organ culture supernatant of biopsies from treated CD patients reflected the presence of positive intestinal TG2 IgA deposits (31).

## Discussion

An extensive review of the literature indicates that intestinal deposits of anti-TG2 IgA are detectable in almost all patients with CD at diagnosis, the only exception being represented by children younger than 2 years of age, where the sensibility of the test is 73%. This data may simply reflect the natural fluctuation of these autoantibodies in serum as previously observed (6). The specificity of this tool varies from 80 to 100% at diagnosis. Control groups were found to show deposits in 5–20% of cases, with the highest prevalence being described in T1D and IBD patients. Both these conditions are well-known autoimmune disorders, characterized by an important role of an environmental component and frequent finding of CD serological markers. Therefore, the presence of intestinal deposits is not entirely surprising in these conditions, and suggests a possible role of gluten as a disease trigger.

No studies specifically investigated the correlation between the presence of intestinal deposits and the clinical presentation of CD. This would be particularly important in the context of the new ESPGHAN diagnostic criteria for CD. According to the new European criteria (33), diagnosis of CD in patients with a classical clinical picture, high antibody titer, and HLA predisposition does not require a SB biopsy. Conversely, children with no clinical symptoms and/or low titer antibodies still need the SB biopsy. In this subgroup of patients, the validation of a further histological marker, i.e., the intestinal TG2 deposits, could be useful.

Results from follow-up studies show that the intestinal deposits slowly disappear after starting treatment with the GFD. In comparison with the serological markers, intestinal deposits tend to disappear later, being still detectable in 56% of patients after many years of GFD (2–42 years). In the same individuals, serological markers were positive only in 15% of patients. Factors influencing the persistence of IgA deposits have not been specifically investigated, particularly we did not find studies correlating the presence of deposits with the adherence to the diet. However, it is interesting to note that the deposits were frequently detectable in a large proportion of patients with non-responsive CD reporting good adherence to the GFD, indicating that in this context their presence may reflect the celiac inflammatory process and not simply the gluten consumption.

Overall few data are available on the possible role of TG2 deposits as an early marker of gluten-exposure during a GC, therefore no conclusion can be drawn on this issue. To test their performance in detecting gluten effects at the intestinal level in CD patients, results from interventional and specifically designed studies (both *in vitro* and *in vivo*) are warranted.

As a marker of early damage in PCD, TG2 deposits were found in a high percentage of patients of all ages at first biopsy, ranging from 68 to 100% of cases. Unfortunately, there are no prospective studies specifically designed to test the sensitivity of intestinal deposits in predicting the development of villous atrophy. Data from a pediatric case-series (39 re-biopsied PCD children) seem to indicate that the finding of TG2 deposits at first observation is a risk factor for developing CD, but these data have never been replicated in larger studies and with a longer period of follow-up. Their application as a predictive tool in PCD continues to be an appealing but still not clearly defined option. In comparison with other methods of intestinal antibodies detection, TG2 deposits were found to be less sensitive and specific than the measurement of anti-TG2 in culture supernatants in PCD at diagnosis (19). Measurement of intestinal antibodies in the culture supernatant is an easier technique, compared to TG2 deposits, and, being an ELISA test, is less influenced by the operator’s ability. Therefore the pros and cons of these two promising tools need to be compared in prospective studies, aiming to characterize the precise role of intestinal antibodies (production and deposition) in predicting villous atrophy.

The TG2 deposits technique has also been used in non-CD conditions, such as other gluten-related disorders and autoimmune conditions. A very high percentage of patients with DH and the totality of a small group with GA showed deposition of TG2 antibodies at the intestinal level. Data from T1D patients are puzzling, considering that 78 and 58% of T1D patients (serologically TG2 positive and negative, respectively) expressed TG2 deposits at the intestinal level. In T1D patients, a transient positivity of serological CD marker is frequently described at diagnosis (34). It is interesting to note that only TG2 deposits in T1D patients with positive serum TTG had the same molecular features of CD deposits. Therefore, it is not clear whether the presence of deposits is a further confirmation of a possible role of gluten in T1D development or is an unspecific autoimmune phenomenon in this group of patients.

It is a limitation of this review that a large proportion of the available data on intestinal deposits come from studies performed by two single European groups, in a relatively short period of time. Confirmation of these results is awaited from other groups and in different settings.

## Conclusion

TG2 deposits are an appealing diagnostic tool, particularly in the setting of challenging CD diagnosis and refractory CD. The value as a prognostic marker in early stage CD has not clearly been established. Overall, the costs and benefits of performing this test need to be very well balanced, considering the technical difficulties and the high costs of the exam.

## Conflict of Interest Statement

The authors declare that the research was conducted in the absence of any commercial or financial relationships that could be construed as a potential conflict of interest.

## References

[1] Fasano A. Clinical presentation of celiac disease in the pediatric population. Gastroenterology (2005) 128(4 Suppl 1):S68–73. 10.1053/j.gastro.2005.02.015 15825129

[2] Marsh MN. Gluten, major histocompatibility complex, and the small intestine. A molecular and immunobiologic approach to the spectrum of gluten sensitivity (‘celiac sprue’). Gastroenterology (1992) 102(1):330–54. 1727768

[3] Collin P Kaukinen K Vogelsang H Korponay-Szabo I Sommer R Schreier E Antiendomysial and antihuman recombinant tissue transglutaminase antibodies in the diagnosis of coeliac disease: a biopsy-proven European multicentre study. Eur J Gastroenterol Hepatol (2005) 17(1):85–91. 10.1097/00042737-200501000-00017 15647647

[4] Scott BB Losowsky MS. Patchiness and duodenal-jejunal variation of the mucosal abnormality in coeliac disease and dermatitis herpetiformis. Gut (1976) 17(12):984–92. 10.1136/gut.17.12.984 1017719 PMC1411232

[5] Tosco A Salvati VM Auricchio R Maglio M Borrelli M Coruzzo A Natural history of potential celiac disease in children. Clin Gastroenterol Hepatol (2011) 9(4):320–5; quiz e36. 10.1016/j.cgh.2010.09.006 20851213

[6] Lionetti E Castellaneta S Pulvirenti A Tonutti E Francavilla R Fasano A Prevalence and natural history of potential celiac disease in at-family-risk infants prospectively investigated from birth. J Pediatr (2012) 161(5):908–14. 10.1016/j.jpeds.2012.05.008 22704250

[7] Shiner M Ballard J Antigen-antibody reactions in jejunal mucosa in childhood coeliac disease after gluten challenge. Lancet (1972) 1(7762):1202–5 10.1016/S0140-6736(72)90924-5 4113189

[8] Korponay-Szabo IR Halttunen T Szalai Z Laurila K Kiraly R Kovacs JB In vivo targeting of intestinal and extraintestinal transglutaminase 2 by coeliac autoantibodies. Gut (2004) 53(5):641–8. 10.1136/gut.2003.024836 15082580 PMC1774023

[9] Walker-Smith JA Guandalini S Schmitz J Shmerling DH Visakorpi JK Revised criteria for diagnosis of coeliac disease. Report of Working Group of European Society of Paediatric Gastroenterology and Nutrition. Arch Dis Child (1990) 65(8):909–11 10.1136/adc.65.8.909 2205160 PMC1792502

[10] Myrsky E Syrjanen M Korponay-Szabo IR Maki M Kaukinen K Lindfors K. Altered small-bowel mucosal vascular network in untreated coeliac disease. Scand J Gastroenterol (2009) 44(2):162–7. 10.1080/00365520802400875 18985542

[11] Oikarinen M Tauriainen S Oikarinen S Honkanen T Collin P Rantala I Type 1 diabetes is associated with enterovirus infection in gut mucosa. Diabetes (2012) 61(3):687–91. 10.2337/db11-1157 22315304 PMC3282798

[12] Salmi TT Collin P Reunala T Maki M Kaukinen K Diagnostic methods beyond conventional histology in coeliac disease diagnosis. Dig Liver Dis (2010) 42(1):28–32 10.1016/j.dld.2009.04.004 19473894

[13] Salmi TT Collin P Jarvinen O Haimila K Partanen J Laurila K Immunoglobulin A autoantibodies against transglutaminase 2 in the small intestinal mucosa predict forthcoming coeliac disease. Aliment Pharmacol Ther (2006) 24(3):541–52. 10.1111/j.1365-2036.2006.02997.x 16886921

[14] Koskinen O Collin P Lindfors K Laurila K Maki M Kaukinen K. Usefulness of small-bowel mucosal transglutaminase-2 specific autoantibody deposits in the diagnosis and follow-up of celiac disease. J Clin Gastroenterol (2010) 44(7):483–8. 10.1097/MCG.0b013e3181b64557 19779364

[15] Koskinen O Collin P Korponay-Szabo I Salmi T Iltanen S Haimila K Gluten-dependent small bowel mucosal transglutaminase 2-specific IgA deposits in overt and mild enteropathy coeliac disease. J Pediatr Gastroenterol Nutr (2008) 47(4):436–42. 10.1097/MPG.0b013e31817b6dec 18852635

[16] Kaukinen K Peraaho M Collin P Partanen J Woolley N Kaartinen T Small-bowel mucosal transglutaminase 2-specific IgA deposits in coeliac disease without villous atrophy: a prospective and randomized clinical study. Scand J Gastroenterol (2005) 40(5):564–72 10.1080/00365520510023422 16036509

[17] Maglio M Tosco A Auricchio R Paparo F Colicchio B Miele E Intestinal deposits of anti-tissue transglutaminase IgA in childhood celiac disease. Dig Liver Dis (2011) 43(8):604–8 10.1016/j.dld.2011.01.015 21342796

[18] Maglio M Tosco A Paparo F Auricchio R Granata V Colicchio B Serum and intestinal celiac disease-associated antibodies in children with celiac disease younger than 2 years of age. J Pediatr Gastroenterol Nutr (2010) 50(1):43–8. 10.1097/MPG.0b013e3181b99c8f 19934769

[19] Tosco A Aitoro R Auricchio R Ponticelli D Miele E Paparo F Intestinal anti-tissue transglutaminase antibodies in potential coeliac disease. Clin Exp Immunol (2013) 171(1):69–75. 10.1111/j.1365-2249.2012.04673.x 23199325 PMC3530097

[20] Kurppa K Ashorn M Iltanen S Koskinen LL Saavalainen P Koskinen O Celiac disease without villous atrophy in children: a prospective study. J Pediatr (2010) 157(3):373–80, 80.e1. 10.1016/j.jpeds.2010.02.070 20400102

[21] Salmi TT Collin P Korponay-Szabo IR Laurila K Partanen J Huhtala H Endomysial antibody-negative coeliac disease: clinical characteristics and intestinal autoantibody deposits. Gut (2006) 55(12):1746–53. 10.1136/gut.2005.071514 16571636 PMC1856451

[22] Tosco A Maglio M Paparo F Rapacciuolo L Sannino A Miele E Immunoglobulin A anti-tissue transglutaminase antibody deposits in the small intestinal mucosa of children with no villous atrophy. J Pediatr Gastroenterol Nutr (2008) 47(3):293–8. 10.1097/MPG.0b013e3181677067 18728524

[23] Koskinen O Villanen M Korponay-Szabo I Lindfors K Maki M Kaukinen K. Oats do not induce systemic or mucosal autoantibody response in children with coeliac disease. J Pediatr Gastroenterol Nutr (2009) 48(5):559–65. 10.1097/MPG.0b013e3181668635 19412007

[24] Koskinen O Lindfors K Collin P Peraaho M Laurila K Woolley N Intestinal transglutaminase 2 specific antibody deposits in non-responsive coeliac disease. Dig Liver Dis (2010) 42(10):692–7. 10.1016/j.dld.2010.03.008 20409763

[25] Salmi TT Hervonen K Laurila K Collin P Maki M Koskinen O Small bowel transglutaminase 2-specific IgA deposits in dermatitis herpetiformis. Acta Derm Venereol (2013). 10.2340/00015555-1764 24352382

[26] Hadjivassiliou M Maki M Sanders DS Williamson CA Grunewald RA Woodroofe NM Autoantibody targeting of brain and intestinal transglutaminase in gluten ataxia. Neurology (2006) 66(3):373–7. 10.1212/01.wnl.0000196480.55601.3a 16476935

[27] Stenberg R Kaukinen K Bengtsson M Lindberg E Dahle C. Early developing celiac disease in children with cerebral palsy. J Pediatr Gastroenterol Nutr (2011) 53(6):674–8. 10.1097/MPG.0b013e318229889d 21697743

[28] Maglio M Florian F Vecchiet M Auricchio R Paparo F Spadaro R Majority of children with type 1 diabetes produce and deposit anti-tissue transglutaminase antibodies in the small intestine. Diabetes (2009) 58(7):1578–84. 10.2337/db08-0962 19401430 PMC2699874

[29] Borrelli M Maglio M Agnese M Paparo F Gentile S Colicchio B High density of intraepithelial gammadelta lymphocytes and deposits of immunoglobulin (Ig)M anti-tissue transglutaminase antibodies in the jejunum of coeliac patients with IgA deficiency. Clin Exp Immunol (2010) 160(2):199–206. 10.1111/j.1365-2249.2009.04077.x 20030673 PMC2857942

[30] Ruuskanen A Luostarinen L Collin P Krekela I Patrikainen H Tillonen J Persistently positive gliadin antibodies without transglutaminase antibodies in the elderly: gluten intolerance beyond coeliac disease. Dig Liver Dis (2011) 43(10):772–8. 10.1016/j.dld.2011.04.025 21641886

[31] Stenman SM Lindfors K Korponay-Szabo IR Lohi O Saavalainen P Partanen J Secretion of celiac disease autoantibodies after in vitro gliadin challenge is dependent on small-bowel mucosal transglutaminase 2-specific IgA deposits. BMC Immunol (2008) 9:6. 10.1186/1471-2172-9-6 18312620 PMC2275217

[32] Tack GJ van de Water JM Bruins MJ Kooy-Winkelaar EM van Bergen J Bonnet P Consumption of gluten with gluten-degrading enzyme by celiac patients: a pilot-study. *World J Gastroenterol* (2013) 19(35):5837–47 10.3748/wjg.v19.i35.5837 PMC3793137 24124328

[33] Husby S Koletzko S Korponay-Szabo IR Mearin ML Phillips A Shamir R European Society for Pediatric Gastroenterology, Hepatology, and Nutrition guidelines for the diagnosis of coeliac disease. J Pediatr Gastroenterol Nutr (2012) 54(1):136–60. 10.1097/MPG.0b013e31821a23d0 22197856

[34] Salardi S Volta U Zucchini S Fiorini E Maltoni G Vaira B Prevalence of celiac disease in children with type 1 diabetes mellitus increased in the mid-1990 s: an 18-year longitudinal study based on anti-endomysial antibodies. J Pediatr Gastroenterol Nutr (2008) 46(5):612–4 10.1097/MPG.0b013e31815d697e 18493223

